# Role of HLA-G and extracellular vesicles in renal cancer stem cell-induced inhibition of dendritic cell differentiation

**DOI:** 10.1186/s12885-015-2025-z

**Published:** 2015-12-24

**Authors:** Cristina Grange, Marta Tapparo, Stefania Tritta, Maria Chiara Deregibus, Antonino Battaglia, Paolo Gontero, Bruno Frea, Giovanni Camussi

**Affiliations:** Department of Medical Sciences, Città della Salute e della Scienza, University of Turin, Corso Dogliotti 14, 10126 Torino, Italy; Translational Center for Regenerative Medicine, Città della Salute e della Scienza, University of Turin, Torino, Italy; Department of Surgical Sciences, Città della Salute e della Scienza, University of Turin, Torino, Italy

**Keywords:** Extracellular vesicles, Exosomes, Microvesicles, Renal cancer stem cells, Immune-escape

## Abstract

**Background:**

Tumor immune-escape has been related to the ability of cancer cells to inhibit T cell activation and dendritic cell (DC) differentiation. We previously identified a tumor initiating population, expressing the mesenchymal marker CD105, which fulfills the criteria for definition as cancer stem cells (CD105^+^ CSCs) able to release extracellular vesicles (EVs) that favor tumor progression and metastases. The aim of the present study was to compare the ability of renal CSCs and derived EVs to modulate the behavior of monocyte-derived DCs with a non-tumor initiating renal cancer cell population (CD105^-^ TCs) and their EVs.

**Methods:**

Maturation of monocyte-derived DCs was studied in presence of CD105^+^ CSCs and CD105^-^ TCs and their derived EVs. DC differentiation experiments were evaluated by cytofluorimetric analysis. T cell proliferation and ELISA assays were performed. Monocytes and T cells were purified from peripheral blood mononuclear cells obtained from healthy donors.

**Results:**

The results obtained demonstrate that both CD105^+^ CSCs and CD105^-^ TCs impaired the differentiation process of DCs from monocytes. However, the immune-modulatory effect of CD105^+^ CSCs was significantly greater than that of CD105^-^ TCs. EVs derived from CD105^+^ CSCs and in less extent, those derived from CD105^-^ TCs retained the ability to impair monocyte maturation and T cell activation. The mechanism has been mainly related to the expression of HLA-G by tumor cells and to its release in a form associated to EVs. HLA-G blockade significantly reduced the inhibitory effect of EVs on DC differentiation.

**Conclusions:**

In conclusion, the results of the present study indicate that renal cancer cells and in particular CSCs and derived EVs impair maturation of DCs and T cell immune response by a mechanism involving HLA-G.

**Electronic supplementary material:**

The online version of this article (doi:10.1186/s12885-015-2025-z) contains supplementary material, which is available to authorized users.

## Background

Tumor is a complex structure constituted by cancer cells with different stages of differentiation. Cancer cells may communicate with surrounding cells to develop an environment favorable to its development. Several studies have suggested that tumor microenvironment may promote oncogenic progression [1]. In renal cell carcinomas, we identified a tumor initiating population, expressing the mesenchymal marker CD105, which fulfills the criteria for definition as cancer stem cells (CSCs) [2]. Namely, this population retains clonogenic ability, expresses Nanog, Nestin and Oct3-4 stem cell markers, and is able to grow in spheres and to induce serially transplantable tumors starting from a number of cells as low as 100 cells [2].

We previously found that extracellular vesicles (EVs) released by renal CD105^+^ CSCs, but not EVs derived from a more differentiated tumor cell population (CD105^-^ TCs), are able to modify tumor microenvironment and to promote development of a lung pre metastatic niche [3].

Several studies indicate that tumor released EVs may orchestrate tumor progression stimulating proliferation, angiogenesis, metastasis formation and immune-escape [4–10]. EVs contain proteins, microRNAs and mRNAs that may be transferred to recipient cells and induce phenotypic and epigenetic changes leading to tumor cell progression and immune-escape [11–14]. In cancer patients the amount of serum-EVs were shown to correlate with a poor prognosis [15]. In addition, tumor-derived EVs, retaining the signature of the cells of origin, may be exploited as a diagnostic tool in cancer [16].

The interaction between tumor and dendritic cells (DCs) has a relevant role in the immune response to tumor antigens. DCs are the most effective antigen presenting cells able to prime naïve T lymphocytes [17]. DCs switch from an immature to an activated state thorough a maturation process. The inhibition of DC maturation has been involved in tumor immune-escape [18]. Several soluble factors have been involved in down regulation of DC activation including IL-6, IL-10, PGE_2_, soluble HLA-G (sHLA-G) and EVs [10, 19–24].

The aim of the present study was to compare the ability of renal CSCs and derived EVs to modulate the behavior of monocyte derived- DCs with a non-tumor initiating renal cancer cell population and their EVs. Herein, we evaluated whether co-incubation with CD105^+^ CSCs and derived EVs interfere with monocyte differentiation into DCs. In fact, immature DCs have been shown to promote induction of tolerance [25].

## Results

### Human CD105^+^ CSCs suppress monocyte-derived DC differentiation and maturation

**Fig. 1:**
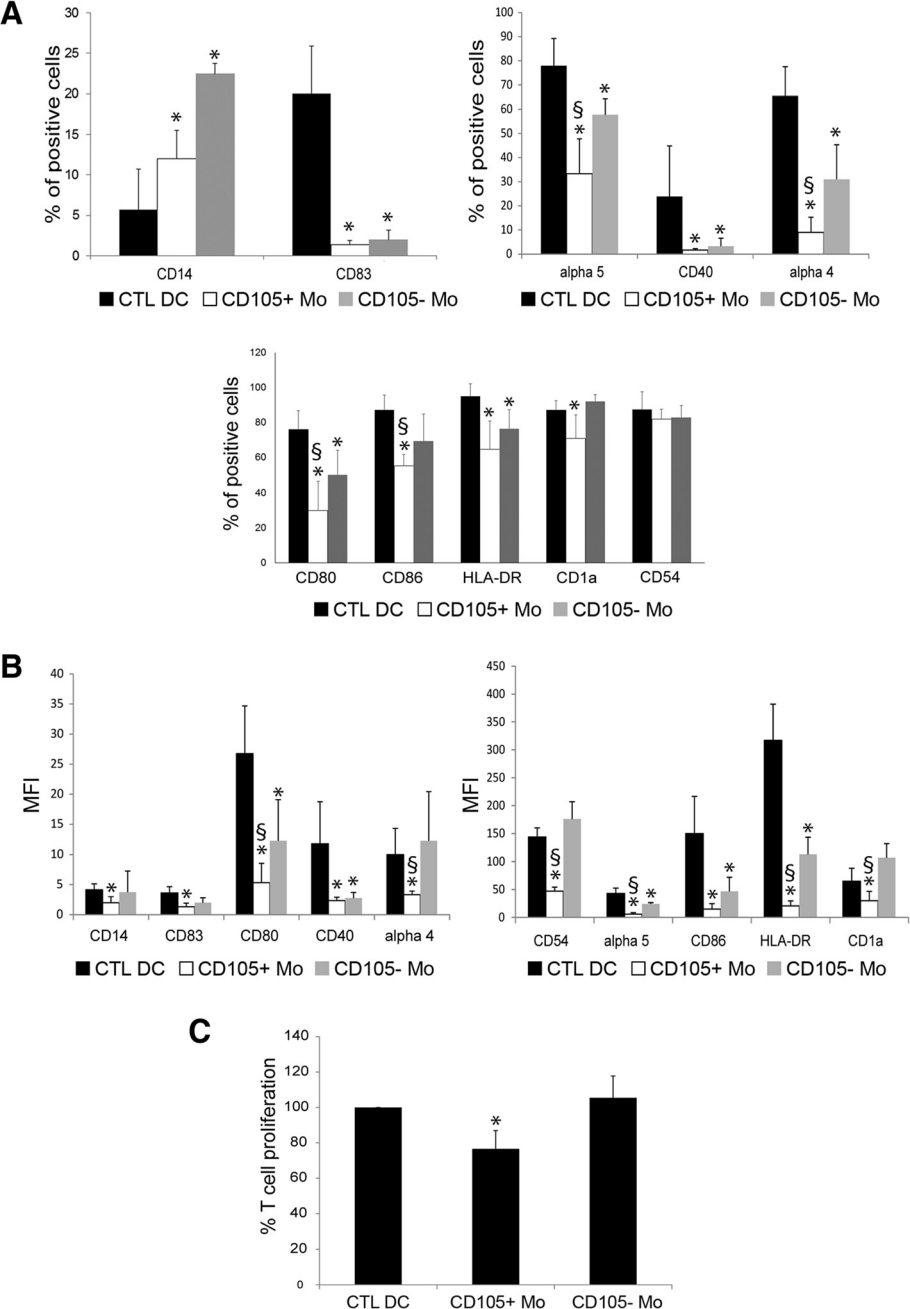
Renal cancer cells suppressed monocyte-derived DC differentiation and their ability to stimulate T cell proliferation. **a** Mean percentage expression ± SD of CD14, CD83, α5 integrin, CD40, α4 integrin, CD80, CD86, HLA-DR, CD1a and CD54 by monocyte-derived DCs differentiated in the presence or in absence (CTL DC) of CD105^+^ CSCs (CD105+ Mo) or CD105^-^ TCs (CD105- Mo). Results were obtained from 6 independent experiments. ANOVA with Newman Keuls multicomparison test was performed: *p < 0.05 CD105+ Mo and CD105- Mo versus CTL DC; § *p* < 0.05 CD105+ Mo versus CD105- Mo. **b** MFI ± SD of CD14, CD83, CD80, CD40, α4 integrin, CD54, α5 integrin, CD86, HLA-DR and CD1a of monocyte-derived DCs differentiated in the presence or in absence (CTL DC) of CD105^+^ CSCs (CD105+ Mo) or CD105^-^ TCs (CD105- Mo). Results were obtained from 6 independent experiments. ANOVA with Newman Keuls multicomparison test was performed: *p < 0.05 CD105+ Mo and CD105- Mo versus CTL DC; § *p* < 0.05 CD105+ Mo versus CD105- Mo. **c** Monocyte-derived DCs differentiated in the presence or in absence (CTL DC) of CD105^+^ CSCs (CD105+ Mo) or CD105^-^ TCs (CD105- Mo) were plated at cell concentration of 2x10^4^ with 1x10^5^ T CD3^+^ lymphocytes. Forty eight hours later T-cell proliferation was assessed. Data are expressed as mean ± SD of percent variation of T-cell proliferation in the presence of DCs differentiated in presence of renal cancer cells in respect to T-cell proliferation in presence of DCs matured in the absence of cells (established as 100 %). Results were obtained from 5 independent experiments. ANOVA with Newman Keuls multicomparison test was performed: *p < 0.05 CD105+ Mo versus all the other conditions

To evaluate inhibition of DC differentiation, monocytes were cultured in the presence of CD105^+^ CSCs and of CD105^-^ TCs. Monocytes, isolated by adhesion (CD14^+^: 87.4 ± 3.5 %, not shown) from Peripheral Blood Mononuclear Cells (PBMCs), were cultured with Granulocyte-Macrophage Colony-Stimulating Factor (GM-CSF) and IL-4 in the presence or absence of CD105^+^ CSCs or CD105^-^ TCs. After 4 days of culture, in order to induce the complete differentiation and maturation of DCs, lipopolysaccharide (LPS) was added. After 7 days, DCs (CTL DC) underwent a complete maturation and acquired their characteristic morphology and appropriate marker expression (Fig. 1a). Properly matured DCs significantly reduced the expression of monocyte/macrophage CD14 marker (5.7 ± 4.9 %) and acquired the expression of CD83 (20.1 ± 5.9 %). Moreover, they increased the expression of CD80, CD86 and HLA-DR (76.1 ± 10.8 %, 87.0 ± 8.7 % and 95.1 ± 7.1 % respectively) and expressed the activation markers CD1a and CD40 (87.9 ± 5.2 %, 23.8 ± 15.5 % respectively) (Fig. 1a).

Co-culture with CD105^+^ CSCs or CD105^-^ TCs interfered with the phenotype of monocyte-derived cells (Fig. 1a). Monocyte-derived cells in the presence of CD105^+^ CSCs (CD105^+^ Mo) or of CD105^-^ TCs (CD105^-^ Mo) maintained CD14 marker (CD105^+^ Mo: 12.0 ± 3.5 % and CD105^-^ Mo: 22.5 ± 1.2 %) and did not acquire activation markers such as CD83 (CD105^+^ Mo: 1.3 ± 0.5 % and CD105^-^ Mo: 2.0 ± 1.1 %) and CD40 (CD105^+^ Mo: 1.6 ± 0.5 % and CD105^-^ Mo: 3.2 ± 3.3 %). Similarly, the antigen-presenting molecule HLA-DR was significantly lower in presence of CD105^+^ CSCs (64.6 ± 16.1 %) and CD105^-^ TCs (76.5 ± 10.8 %) in comparison with control DCs. However, the inhibitory effect was significantly higher in presence of CD105^+^ CSCs than CD105^-^ TCs. The percentage of monocyte-derived cells positive for costimulatory molecules CD80 (CD105^+^ Mo: 30.0 ± 16.0 % and CD105^-^ Mo: 50.2 ± 13.8 %) and CD86 (CD105^+^ Mo: 55.5 ± 6.4 % and CD105^-^ Mo: 69.5 ± 15.4 %) and for the specific dendritic marker CD1a (CD105^+^ Mo: 71.0 ± 13.2 % and CD105^-^ Mo: 92.0 ± 4.0 %) were significantly reduced by CD105^+^ CSCs compared to CD105^-^ TCs. Both CD105^+^ CSCs and CD105^-^ TCs significantly reduced also the expression of adhesion molecules such as integrin α4 and α5, involved in T cell contact, by monocyte-derived cells (Fig. 1a). CD105^+^ CSCs were significantly more effective than CD105^-^ TCs in reducing the percentage of integrin α4 and α5 positive cells on monocyte-derived cells (integrin α4: CD105^+^ Mo: 9.0 ± 6.2 % and CD105^-^ Mo: 31.0 ± 14.3 %; integrin α5: CD105^+^ Mo: 33.3 ± 14.4 % and CD105^-^ Mo: 57.7 ± 6.6 %). The mean fluorescence intensity (MFI) confirmed the inhibitory effect of CD105^+^ CSCs in respect to control DCs (Fig. 1b and Additional 1: Table S1). The inhibitory effect of CD105^-^ TCs was significant only for some markers (CD80, CD86, CD40, HLA-DR and α5 integrin). When CD105^+^ CSCs were compared with CD105^-^ TCs, MFI of monocyte-derived cells was significantly reduced for costimulatory, activation and adhesion molecules such as CD14, CD83, CD80, HLA-DR, CD1a, α4 integrin and CD54 (Fig. 1b and Additional 1: Table S1).

### Monocyte-derived cells cultured with CD105^+^ CSCs failed to induce T cell proliferation

**Fig. 2:**
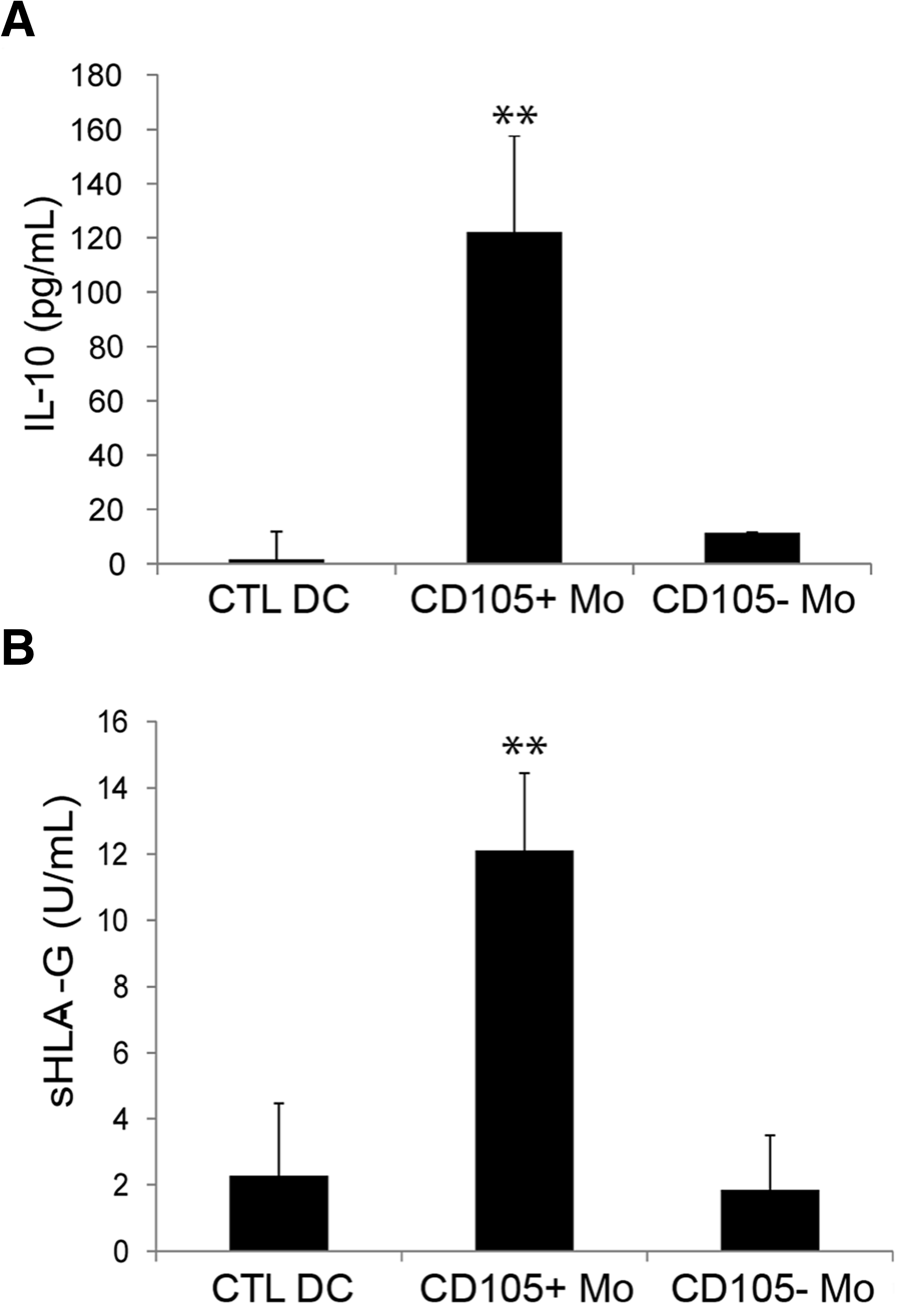
Co-culture of monocyte-derived cells with CD105^+^ CSCs induced the release of IL-10 and sHLA-G. Cell supernatants were harvested to detect IL-10 (**a**) and sHLA-G (**b**) production by ELISA, after 7 days of co-culture of monocyte-derived cells in the presence or absence (CTL DC) of renal cancer cells (CD105^+^ CSCs and CD105^-^ TCs). Results were obtained from 3 independent experiments and expressed as mean ± SD. ANOVA with Newman Keuls multicomparison test was performed: ** *p* < 0.001 CD105^+^ CSC Mo versus all the other conditions

Mature DCs were the main antigen presenting cells and were able to induce T cell response and activation. LPS-stimulated DCs were capable to induce CD3^+^ lymphocyte proliferation. The preconditioning of monocyte-derived cells by co-culture with CD105^+^ CSCs significantly impaired their ability to stimulate CD3^+^ lymphocyte proliferation (Fig. 1c). The co-culture of monocyte-derived cells with CD105^+^ CSCs induced the release of IL-10 soluble factor (122.5 ± 25.6 pg/ml) (Fig. 2a). On the contrary, the co-culture with CD105^-^ TCs induced only a slight increase of IL-10 production (11.3 ± 0.5 pg/ml) (Fig. 2a).

### sHLA-G was released in cell free supernatant of monocyte-derived cells co-cultured with CD105^+^ CSCs

**Fig. 3:**
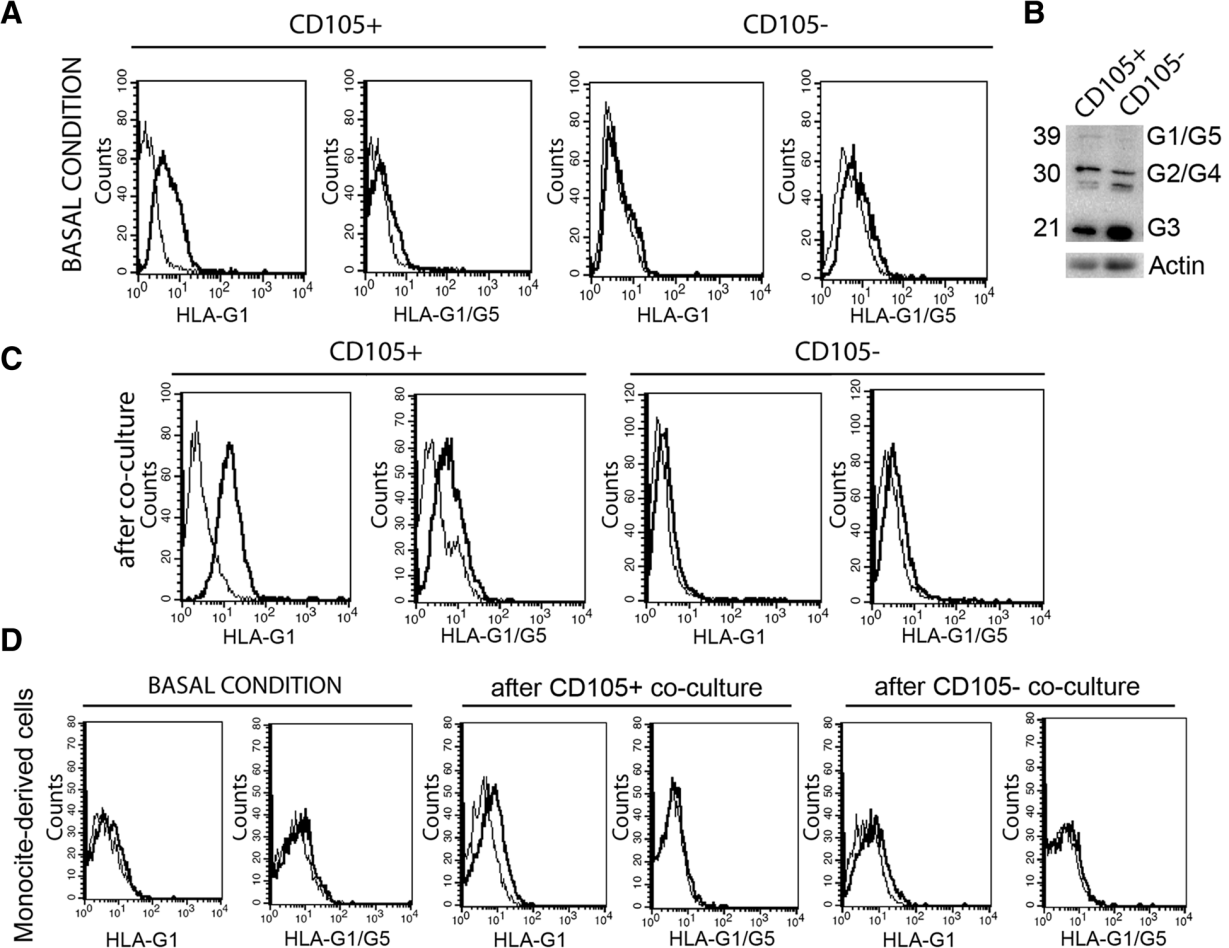
HLA-G expression and up-regulation by CD105^+^ CSCs. **a** Representative cytofluorimetric analysis of HLA-G expression on CD105^+^ CSCs and CD105^-^ TCs membrane (G1) and intra-cytoplasmic (G1/G5) staining in basal culture condition (n = 4). **b** Western Blot analyses confirmed the presence of several isoforms of HLA-G in CD105^+^ CSCs and CD105^-^ TCs. **c** Representative cytofluorimetric analysis of membrane (G1) and intra-cytoplasmic (G1/G5) staining of HLA-G on CD105^+^ CSCs and CD105^-^ TCs after 7 days of co-culture with monocyte-derived cells (n = 4). **d** Representative cytofluorimetric analysis of HLA-G expression on monocyte-derived cell membrane (G1) and intra-cytoplasmic (G1/G5) in basal condition and after co-culture with CD105^+^ CSCs and CD105^-^ TCs (n = 4)

The non-classical human leukocyte antigen HLA-G was evaluated. By ELISA assay, the soluble HLA-G (sHLA-G) was barely detectable in cell free supernatants of renal cancer cells (not shown) and of fully differentiated DCs (Fig. 2b). On the contrary, the level of sHLA-G was significantly increased in supernatant of monocyte-derived cells co-cultured with CD105^+^ CSCs (12.1 ± 2.3 U/ml) compared with CD105^-^ TCs (1.8 ± 1.6 U/ml) or with control DCs (2.2 ± 2.1 U/ml) (Fig. 2b). The expression of HLA-G by renal cell carcinomas and the presence of its soluble form in plasma of patients were previously described [26, 27]. CD105^+^ CSCs in basal condition expressed the membrane bound isoform HLA-G1 composed by 3 globular domains. In CD105^-^ TCs, the isoform HLA-G1 was barely detectable (Fig. 3a and b). The cytoplasmic isoform HLA-G1/G5 was expressed at very low level by both CD105^+^ CSCs and by CD105^-^ TCs (Fig. 3a). When CD105^+^ CSCs were co-cultured in transwell for 1 week with monocyte-derived cells, they up regulated the expression of HLA-G1 isoform as well as of the cytoplasmic isoform HLA-G1/G5 (Fig. 3c). No significant up-regulation was observed in CD105^-^ TCs co-cultured with monocyte-derived cells. On the other hand, monocyte-derived cells showed a slight increase of HLA-G1 after co-culture with CD105^+^ CSCs (Fig. 3d). These data suggest that the presence of sHLA-G in the supernatant of co-culture is mainly due to secretion by CD105^+^ renal CSCs.

### EVs contributed to the inhibitory effect of renal CSCs on monocyte-derived cell differentiation by carrying HLA-G

**Fig. 4:**
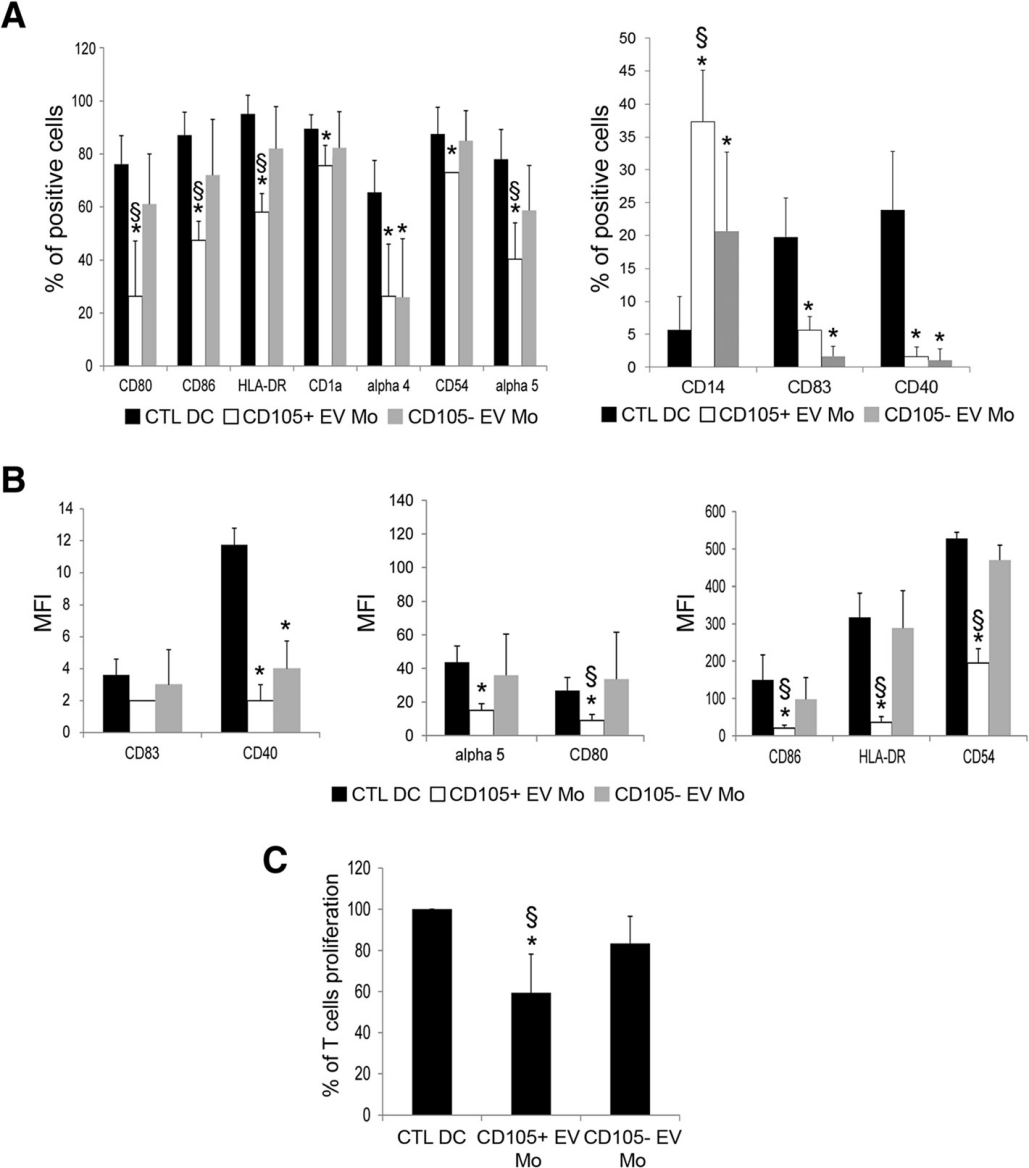
EVs shed by renal cancer cells inhibited monocyte-derived DC differentiation and their ability to stimulate T cell proliferation. **a** Mean percentage expression ± SD of CD80, CD86, HLA-DR, CD1a, α4 integrin, CD54, α5 integrin, CD14, CD83 and CD40 by monocyte-derived DCs differentiated in the presence or in absence (CTL DC) of CD105^+^ EVs (CD105+ EV Mo) or CD105^-^ EVs (CD105- EV Mo). Results were obtained from 6 independent experiments. ANOVA with Newman Keuls multicomparison test was performed: *p < 0.05 CD105+ EV Mo and CD105- EV Mo versus CTL DC; § *p* < 0.05 CD105+ EV Mo versus CD105- EV Mo. **b** MFI ± SD of CD83, CD40, α5 integrin CD80, CD86, HLA-DR and CD54 of monocyte-derived DCs differentiated in the presence or in absence (CTL DC) of CD105^+^ EVs (CD105+ EV Mo) or CD105^-^ EVs (CD105- EV Mo). Results were obtained from 6 independent experiments. ANOVA with Newman Keuls multicomparison test was performed: *p < 0.05 CD105+ EV Mo and CD105- EV Mo versus CTL DC; § *p* < 0.05 CD105+ EV Mo versus CD105- EV Mo. **c** Monocyte-derived DCs differentiated in the presence or in absence (CTL DC) of CD105^+^ EVs (CD105+ EV Mo) or CD105^-^ EVs (CD105- EV Mo) were plated at cell concentration of 2x10^4^ with 1x10^5^ T CD3^+^ lymphocytes. Forty eight hours later T-cell proliferation was assessed. Data are expressed as mean ± SD of percent variation of T-cell proliferation in the presence of DCs differentiated in the presence of renal cancer cells in respect to T-cell proliferation in presence of DCs matured in the absence of EVs (established as 100 %). Results were obtained from 4 independent experiments. ANOVA with Newman Keuls multicomparison test was performed: *p < 0.05 CD105+ EV Mo versus all the other conditions

As shown in Fig. 4a, both EVs derived from CD105^+^ CSCs and CD105^-^ TCs interfered with the DC differentiation process. Stimulation with EVs significantly abrogated the expression of activation markers CD83 (CD105^+^ EV Mo: 5.6 ± 2.0 % and CD105^-^ EV Mo: 1.6 ± 1.5 %) and CD40 (CD105^+^ EV Mo: 1.6 ± 1.5 % and CD105^-^ EV Mo: 1.0 ± 1.7 %) by monocyte-derived cells. At the same time, monocyte-derived cells maintained CD14 expression (CD105^+^ EV Mo: 37.3 ± 7.7 % and CD105^-^ EV Mo: 20.6 ± 12 %) (Fig. 4a). However, the inhibitory effect of EVs derived from CD105^+^ CSCs was significantly greater than that of EVs derived from CD105^-^ TCs with particular regard to the maintenance of CD14 expression*.* Stimulation with CD105^+^ EVs, but not with CD105^-^ EVs, strongly reduced the costimulatory molecules such as CD80 (CD105^+^ EV Mo: 26.3 ± 20.7 % and CD105^-^ EV Mo: 61.3 ± 19.1 %) and CD86 (CD105^+^ EV Mo: 47.3 ± 7.2 % and CD105^-^ EV Mo: 72.0 ± 21.4 %) and the antigen presenting molecule HLA-DR (CD105^+^ EV Mo: 58.3 ± 7.0 % and CD105^-^ EV Mo: 82.2 ± 15.8 %) on monocyte-derived cells compared with DCs (CTL DC) (Fig. 4a). Furthermore, the inhibitory effect of CD105^+^ EVs was evident also on the reduction of adhesion molecule CD54 (CD105^+^ EV Mo: 73.2 ± 20.7 % and CD105^-^ EV Mo: 85.3 ± 11.3 %) and α5 integrin (CD105^+^ EV Mo: 40.3 ± 13.6 % and CD105^-^ EV Mo: 58.6 ± 17.2 %) on monocyte-derived cells (Fig. 4a).

The interference of DC differentiation and maturation process induced by CD105^+^ EVs appeared also clear by analysing the fluorescence intensity expressed as MFI (Fig. 4b and Additional 2: Table S2). CD105^+^ EVs significantly reduced the MFI of CD40, α5 integrin, CD80, CD86, HLA-DR and CD54 on monocyte-derived cells compared with CD105^-^ EVs or with control DCs (Fig. 4b and Additional 2: Table S2).

DCs differentiated in the presence of EVs shed by CD105^+^ CSCs failed to induce T cell proliferation (Fig. 4c). The pretreatment of monocyte-derived cells with CD105^+^ EVs significantly impaired the ability of these cells to stimulate CD3^+^ lymphocyte proliferation (Fig. 4c). Monocyte-derived cells stimulated with CD105^+^ EVs and CD105^-^ EVs released significant amount of IL-10 (191.6 ± 91.1 pg/ml for CD105^+^ EVs and 141 ± 70.3 pg/ml for CD105^-^ EVs) compared with control DCs (1.7 ± 10.1 pg/ml).

### The involvement of HLA-G carried by EVs on the inhibitory effect of CD105^+^ EVs on monocyte-derived DC differentiation

**Fig. 5:**
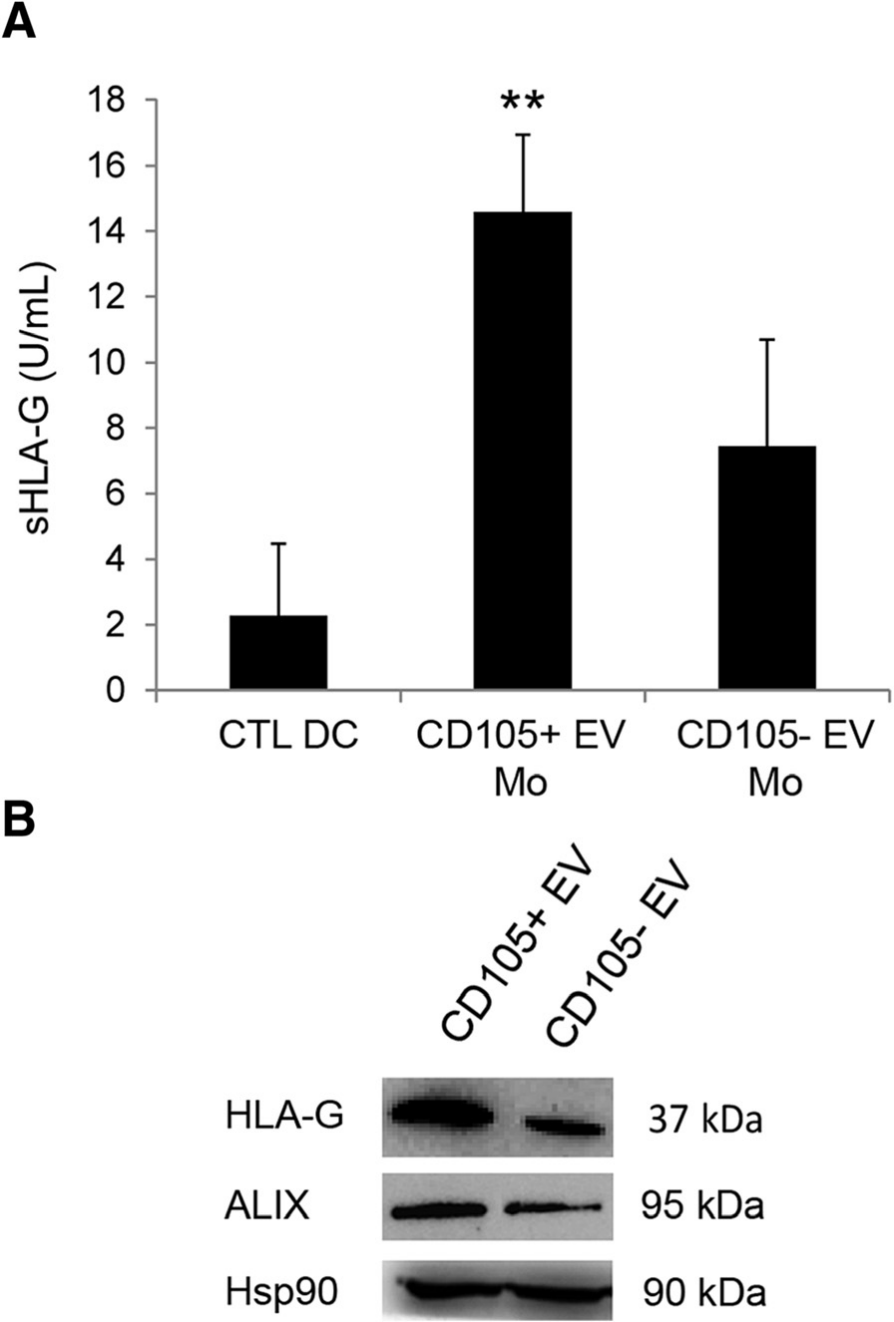
Treatment of monocyte-derived cells with CD105^+^ EVs induced a release of sHLA-G. **a** Supernatants were harvested to detect sHLA-G production by ELISA, after 7 days of culture of monocyte-derived cells stimulated with EVs shed by renal cancer cells (CD105^+^ CSCs and CD105^-^ TCs). Results were obtained from 3 independent experiments and expressed as mean ± SD. ANOVA with Newman Keuls multicomparison test was performed: ** *p* < 0.001 CD105+ EV Mo versus all the other conditions. **b** Representative Western Blot analysis showing the presence of HLA-G and Alix within EVs. Hsp90 was used as normalization. Four experiments were performed with similar results

The level of sHLA-G was tested on supernatant of monocyte-derived cells stimulated with EVs. Monocyte-derived cells treated with CD105^+^ EVs showed the presence of sHLA-G in the supernatant of culture after 7 days (14.5 ± 2.3 U/ml) (Fig. 5a); a lower level of sHLA-G was observed using CD105^-^ EVs as stimulus (7.4 ± 3.2 U/ml).

The presence of HLA-G within EVs was demonstrated by Western Blot (Fig. 5b); both CD105^+^ EVs and CD105^-^ EVs carried HLA-G. The amount was greater in EVs shed by CD105^+^ CSCs than by CD105^-^ TCs (Fig. 5b).

**Fig. 6:**
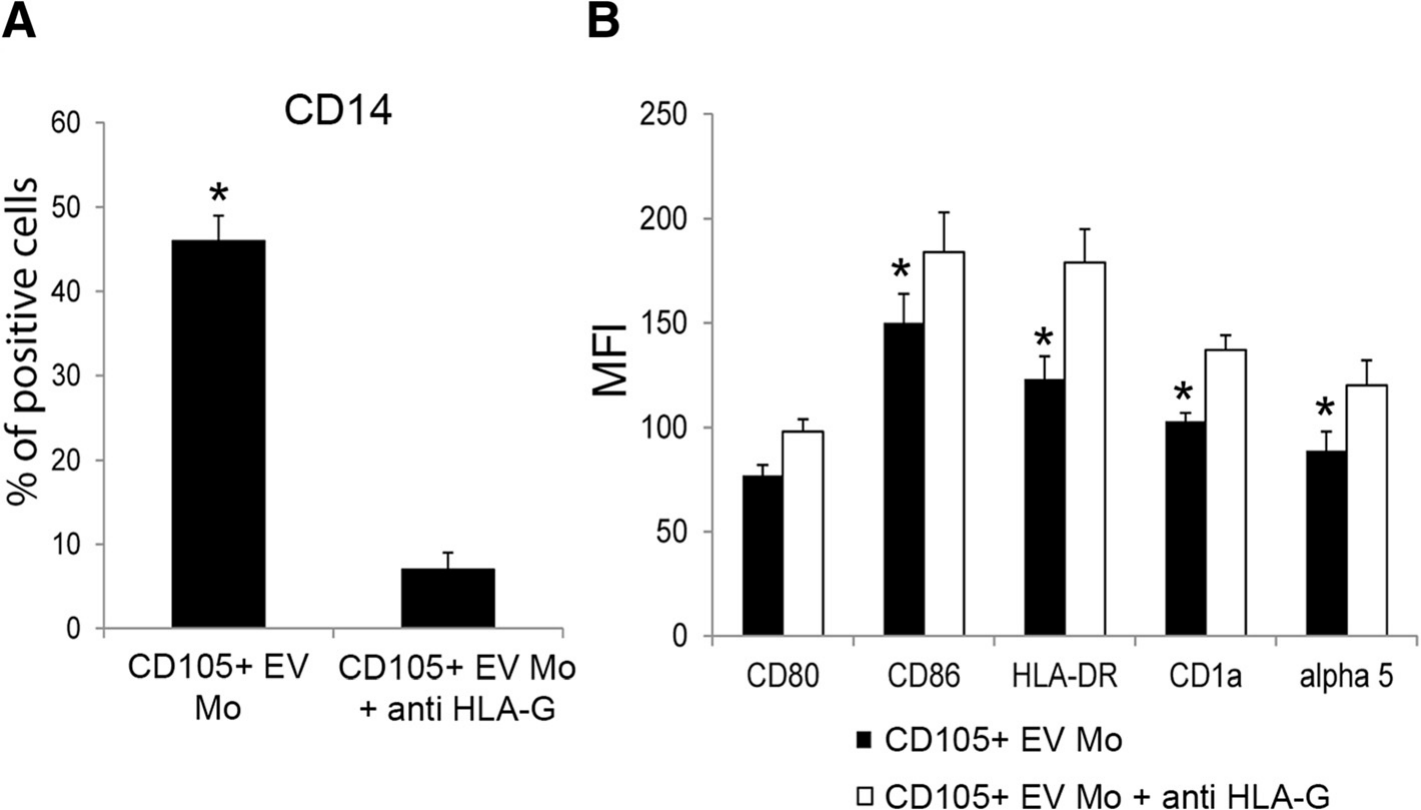
The use of specific blocking antibody against HLA-G partially reverted the inhibitory effect on monocyte-derived DC differentiation induced by CD105^+^ CSC EVs. **a** Mean percentages ± SD of CD14 expression by monocyte-derived DCs stimulated with CD105^+^ EVs in the presence or in absence (CD105^+^ EV Mo) of specific blocking antibody against HLA-G (CD105^+^ EV Mo + anti HLA-G). Results were obtained from 2 independent experiments. T Student test was performed: *p < 0.05 CD105^+^ EV Mo versus CD105^+^ EV Mo + anti HLA-G. **b** MFI ± SD of CD80, CD86, ΗLA-DR, CD1a and α5 integrin of monocyte-derived DCs stimulated with CD105^+^ EVs in the presence or in absence (CD105^+^ EV Mo) of specific blocking antibody against HLA-G (CD105^+^ EV Mo + anti HLA-G). T Student test was performed: *p < 0.05 CD105^+^ EV Mo versus CD105^+^ EV Mo + anti HLA-G

To demonstrate a relevant role of sHLA-G in the monocyte-derived DC differentiation process, a blocking antibody was added to monocyte-derived cells plus CD105^+^ EVs. The presence of blocking antibody partially reverted the inhibitory effect of EVs shed by CD105^+^ CSCs (Fig. 6). It was observed that anti-HLA-G antibody abrogated the maintenance of the monocyte/macrophage marker CD14 induced by CD105^+^ EVs on monocyte-derived cells (CD105^+^ EV: 46.4 ± 3.0 % and CD105^+^ EV + anti-HLA-G: 7.5 ± 2.1 %) (Fig. 6a). In addition, the anti-HLA-G antibody significantly reverted the MFI reduction of CD86 (CD105^+^ EV: 150 ± 14 % and CD105^+^ EV + anti-HLA-G: 184 ± 19 %), HLA-DR (CD105^+^ EV: 123 ± 11 % and CD105^+^ EV + anti-HLA-G: 179 ± 16 %), CD1a (CD105^+^ EV: 103 ± 4 % and CD105^+^ EV + anti-HLA-G: 137 ± 7 %) and α5 integrin (CD105^+^ EV: 89 ± 9 % and CD105^+^ EV + anti-HLA-G: 120 ± 12 %) on monocyte-derived cells (Fig. 6b).

## Discussion

Monocyte-derived DCs are considered the end differentiation step of blood monocytes. This process requires transition from an immature to a mature stage and has a pivotal role in initiating immunity. During this process, DCs up regulate many surface molecules such as costimulatory and activator molecules [19]. The principal role of mature DCs is the antigen presentation and the activation of naïve T lymphocytes [28].

The results of the present study demonstrate that renal cancer cells impaired the differentiation process of DCs from monocytes. Several soluble factors including cytokines and chemokines may influence DC phenotype [19–24]. Here we demonstrated a contribution of EVs in this process. Monocytes co-cultured with CD105^+^ CSCs or CD105^-^ TCs did not up regulate the antigen-presenting molecule HLA-DR and did not acquire activation markers such as CD83 and CD40, expressed by appropriately differentiated and matured DCs. The immune-modulatory effect of CD105^+^ CSCs was significantly more efficient than that of CD105^-^ TCs. In particular, the presence of CD105^+^ CSCs reduced the acquisition of the specific dendritic marker CD1a and down regulated the expression of costimulatory molecules (CD80 and CD86) and of adhesion molecules (α4 and α5 integrin and CD54).

Strong evidence indicates that tumor growth is not just determined by malignant cancer cells, but requires a favourable microenvironment generated by many non-tumor cells such as fibroblasts, endothelial cells and immune cells [29]. Recent studies have suggested that tumour cells possess strong capacity to modulate the immune-response by inhibition of T cells, NK response and DC differentiation and by promoting myeloid derived suppressor cells with T cell inhibitory capacity [30–32]. It is generally recognized that tumors contain heterogeneous populations of tumor cells with different proliferation and differentiation potential [33, 34]. The relative contribution of these populations to immune-suppression has not yet been completely defined. We herein demonstrated that the CD105^+^ CSC population was the main responsible for making monocyte-derived cells unable to stimulate T cell proliferation.

Cancer cells are known to release a wide range of soluble factors in order to promote tumor progression. Previous studies demonstrated that tumor cells were able to shed large amount of EVs [35] that may act on neighbouring or distant cells changing their phenotype [36–40]. EVs released from CSCs were found to modify the tumor microenvironment promoting vascularization and inducing lung pre-metastatic niche formation [3]. Moreover, we recently found that CSC-derived EVs induced a pro-tumorigenic phenotype in mesenchymal stromal cells [41].

In the present study we demonstrated that CSC derived EVs are the main mediators of the inhibitory effect on monocyte differentiation and maturation into DCs. EVs shed from CD105^+^ CSCs inhibited DC differentiation process whereas EVs derived from CD105^-^ TCs only partially interfered with DC differentiation. In the presence of CD105^+^ EVs, monocyte-derived cells retained the monocyte/macrophage CD14 marker and strongly reduced the expression of HLA-DR, costimulatory molecules and adhesion molecules. In addition, DCs differentiated in the presence of CD105^+^ and not CD105^-^ EVs, significantly reduced proliferation of CD3 lymphocytes.

HLA-G carried by EVs was shown to contribute to this inhibitory effect. HLA-G is a non-classical HLA-class I molecule with a low polymorphism and a restricted tissue distribution. Originally, HLA-G was described to protect the fetus against the maternal immune system [42]. HLA-G was shown to suppress the function of NKs, T cells and DCs [43]. Moreover, HLA-G is also involved in cancer immune-escape [44–47]. In particular, HLA-G has been shown to be up-regulated in 50 % of clear cell renal cell carcinomas and the presence of its soluble form has been detected in plasma of these patients [26, 27]. CD105^+^ CSCs expressed high level of HLA-G compared with CD105^-^ TCs. The co-culture with immune cells significantly increased HLA-G expression by CD105^+^ CSCs as well as the soluble form. We also found that sHLA-G was mainly associated with EVs released from CD105^+^ CSCs and in less extent with those released from CD105^-^ TCs. Experiments with anti-HLA-G blocking antibody demonstrated that the inhibitory effect of CD105^+^ EVs on monocyte maturation was significantly reduced suggesting that EV-carried HLA-G plays a relevant immune-modulatory role.

## Conclusions

In conclusion, the results of the present study indicate that renal cancer cells and derived EVs impair maturation of DCs and T cell immune response. This effect was mainly ascribed to the CSC population and to the release of HLA-G-carrying EVs.

## Methods

The study has been approved by the ethic Committee of the Azienda Ospedaliera Universitaria, Città della Salute e della Scienza, Torino (N. 168/2014).

### Renal cancer cells

CD105^+^ CSC clones and CD105^**-**^ TCs were previously isolated and characterized [2] and were cultivated in DMEM LG (Invitrogen, Paisly, UK), with insulin-transferrin-selenium, 10^-9^ M dexametasone, 100 U penicillin, 1000 U streptomycin, 10 ng/ml Epidermal Growth Factor (all from Sigma-Aldrich, St. Louis, MO, USA) and 5 % FCS (EuroClone, Wetherby, UK). To avoid the presence of non-neoplastic contaminating cells, CD105^+^ CSCs were grown in expansion medium without serum or were cloned. Three clones originating from 3 different renal cell carcinomas were used. The CD105^-^ population was unable to generate clones [2]. CD105^+^ CSCs expressed the CD105 mesenchymal marker and other mesenchymal stromal cell markers such as CD90, CD44, CD73, CD29, CD146 and vimentin. Additionally they were positive for the embryonic renal marker Pax2 as well as Nanog, Oct3-4, Musashi, and Nestin. They were clonogenic and generated spheres in non-adhesive cell culture medium. CD105^+^ CSCs displayed the ability to initiate tumors and generate serially transplantable tumors with a number of cells as low as 100 cells/mouse recapitulating the histological pattern of the tumor of origin [3, 41]. As previously described, CD105^+^ CSCs were negative for endothelial or haematopoietic markers [2, 3, 41].

### EV isolation and characterization

EVs were obtained from cell supernatants by ultracentrifugation as previously described [3]. In brief, CD105^+^ CSCs and CD105^-^ TCs were cultured overnight in RPMI (Euroclone). Cell supernatant was centrifuged twice at 3000 g for 10 min to remove cell debris and then ultracentrifuged at 100.000 g (Beckman Coulter Optima L-90 K ultracentrifuge, Brea, CA, USA) for 2 h at 4 °C. EVs were stored in serum free RPMI supplemented with 10 % DMSO at -80 °C. EV number was quantified by NanoSight LM10 instrument (NanoSight Ltd., Amesbury, UK) equipped with the nanoparticle tracking analysis (NTA) 2.0 analytic software. The protein content of EV preparations was quantified by Quantifluor (Promega) using NanoOrange Protein Quantitation Kit (Life Technologies, Carlsbad, CA). Cytofluorimetric analysis was performed by Guava easyCyte Flow Cytometer (Millipore, Billerica, MA, USA) and analyzed with InCyte software using the following FITC- or PE- conjugated antibodies: CD44, CD105, α5 integrin, α6 integrin (Miltenyi Biotec, Bergisch Gladbach, Germany), CD73, CD29, CD90 and CD146 (BD Biosciences) [48]. FITC or PE mouse isotypic IgG (Miltenyi Biotec) were used as control (Additional 3: Figure S1).

### Peripheral blood mononuclear cell isolation

PBMCs were obtained from healthy donors (n = 5 males; n = 9 females; age 25-50 years old). All subjects gave informed consent and the study was approved by the internal Review Board of Blood Bank, Azienda Ospedaliera Universitaria, Città della Salute e della Scienza. PBMCs were isolated by centrifugation over Histopaque-1077 (Sigma).

### DC differentiation

Monocytes were isolated by plastic adherence. PBMCs were plated at the concentration of 5x10^6^ cells/ml in DC differentiation medium, composed by RPMI supplemented with 10 % FCS, 20 ng/ml of IL-4 (Sigma) and 50 ng/ml of GM-CSF (Sigma), in 6-well flat-bottomed plates, and left to adhere overnight [49]. Non-adherent cells were removed the day after and the purity of monocyte population was quantified by positive staining for CD14 (CD14^+^: 87.4 ± 3.5 %, not shown), assessed by FACS analysis. Monocytes were co-cultured in the presence of 1x10^5^ CD105^+^ CSCs or CD105^-^ TCs seeded in transwell (1 μm-pore) or with the amount of EVs released by the same number of cells. CD105^+^ CSCs release a mean of 913 ± 40 particles/cell and CD105^-^ TCs shed a mean of 1075 ± 65 particles/cell, so we stimulated monocytes with 0.9x10^8^ CD105^+^ EVs and with 1.0x10^8^ CD105^-^ EVs. After 2 days, 1/3 of the medium was replaced and after 4 days, 200 ng/ml of LPS (Sigma) was added to the culture. At this time, renal cancer cells were replaced (1x10^5^ cells/transwell) and the stimulus with EVs was added again. Complete differentiation was reached after 7 days. The DC differentiation was assessed after 7 days of culture using FITC, PE and APC conjugated antibodies (all from Becton Dickinson Bioscence, San Josè, CA) for CD14, CD80, CD86, HLA-DR, CD1a, CD83, CD40, CD54, α4 and α5 integrin. Data were expressed as percentage of positive cells ± SD and as geometric mean of MFI ± SD.

In selected experiments, to test the functional role of HLA-G, the differentiation of monocytes was performed in presence of CD105^+^ EVs plus the specific neutralizing antibody HLA-G (clone 87-G, EXBIO, Praha, Czech Republic) at the concentration of 10 μg/ml added twice.

### T cell isolation and proliferation

CD3 positive lymphocytes were sorted from PBMCs using anti-CD3 magnetic beads (Miltenyi Biotec). T cells were used for proliferation assay. Completely differentiated DCs in the presence or not of tumor cells or EVs were treated with 50 μg/ml mitomycin C (Sigma) in order to block their proliferation and cultured in triplicate in a concentration of 2x10^4^ in 96 well flat-bottomed plate with 1x10^5^ allogeneic CD3^+^ lymphocytes [21]. As positive control CD3^+^ cells were treated with 10 ng/ml of PMA (phorbol 12-myristate 13-acetate, Sigma). Proliferation rate was analyzed after 3 days of co-culture using 5-bromo-2’-deoxy-uridine (BrdU) incorporation kit (Roche, Basel, Switzerland). Optical density was measured with an ELISA reader at 405 nm. Data are expressed as mean ± SD of percent variation of T-cell proliferation in the presence of DCs differentiated with renal cancer cells in respect to T-cell proliferation induced by DCs matured in the absence of cells (established as 100 %).

### ELISA assays

sHLA-G and IL-10 were quantified in the cell supernatants of monocyte-derived cell culture in absence or in presence of CD105^+^ CSCs and CD105^-^ TCs by enzyme-linked immunosorbent assay (ELISA, R&D System, Minneapolis, MN).

### Detection of HLA-G by FACS

The expression of HLA-G, (dilution 1:20) on renal cancer cells and on monocyte-derived cells was evaluated by FACS analysis using the specific MEMG/11 antibody (native form for human HLA-G1, EXBIO, Praha, Czech Republic) and MEMG/9 (native form for human HLA-G1 and for soluble HLA-G5 EXBIO, Praha, Czech Republic). Extracellular and intra-cellular staining were used to study membrane bound and intra-cytoplasmatic HLA-G protein.

### Western Blot

HLA-G, ALIX and Hsp90 expression was analyzed by Western Blot. In brief, cells and EVs were lysed in RIPA buffer containing a cocktail of protease inhibitors and the quantification was performed using Bradford. EV proteins or total cell lysates were solubilized in Laemli sample buffer at 95 °C, under reducing conditions and separated in 4-15 % sodium dodecyl sulfate (SDS)-polyacrylamide gel electrophoresis. Nitrocellulose membranes (Bio-Rad laboratories) were blotted with antibodies specific for HLA-G (1:200), Hsp90 (1:200) and ALIX (1:200) (from Santa Cruz Biotechnology, Santa Cruz CA) and with anti-mouse or anti-rabbit horseradish peroxidase (HRP)-conjugated antibodies (1:3000) (Pierce, Waltham, MA) and developed using ECL plus detection reagents (GE Healthcare, Piscataway, NJ). Densitometry analysis was performed using Quantity One image acquisition and analysis software (Bio-Rad laboratories).

### Statistical analysis

Results are expressed as mean ± SD. Statistical analysis was performed by using the *t* test, ANOVA with Newmann-Keuls, or ANOVA with Dunnet’s multicomparison tests when appropriate. A *p* value of <0.05 was considered significant.

## Additional files

Additional 1: Table S1. Mean Fluorescence Intensity (MFI) of monocyte-derived cells cultured in presence or absence of renal cancer cells (CD105^+^ CSCs and CD105^-^ TCs). (DOCX 13 kb) Additional 2: Table S2. Mean Fluorescence Intensity (MFI) of monocyte-derived cells stimulated with or without EVs shed by CD105^+^ CSCs and CD105^-^ TCs. (DOCX 14 kb) Additional 3: Figure S1. EVs characterization. A. Representative size distribution of EVs shed by CD105^+^ CSCs and CD105^-^ TCs obtained using NanoSight LM10 instrument equipped with the nanoparticle tracking analysis (NTA) 2.0 analytic software. B. Representative cytofluorimetric analysis performed by Guava easyCyte Flow Cytometer of EVs shed by CD105^+^ CSCs and CD105^-^ TCs and analyzed with InCyte software. The following markers were evaluated: CD44, CD105, α5 integrin, α6 integrin, CD73, CD29, CD90 and CD146. (DOCX 451 kb)

## Abbreviations

CSC: Cancer stem cell
TC: Tumor cell
DC: Dendritic cell
EV: Extracellular vesicle
PBMC: Peripheral blood mononuclear cell
PMA: Phorbol 12-myristate 13-acetate
GM-CSF: Granulocyte-Macrophage Colony-Stimulating Factor
MFI: Mean fluorescence intensity
LPS: Lipopolysaccharide
