# Supplementary material for: Role of HLA-G and extracellular vesicles in renal cancer stem cell-induced inhibition of dendritic cell differentiation
Source: BMC Cancer. 2015 Dec 24;15:1009. doi: 10.1186/s12885-015-2025-z (PMC4690241; doi:10.1186/s12885-015-2025-z)
Supplement: Additional 1: Table S1. — Mean Fluorescence Intensity (MFI) of monocyte-derived cells cultured in presence or absence of renal cancer cells (CD105+ CSCs and CD105- TCs). (DOCX 13 kb) [file 12885_2015_2025_MOESM1_ESM.docx]

**Additional Table 1**

**Mean Fluorescence Intensity (MFI) of monocyte-derived cells cultured in presence or absence of renal cancer cells (CD105^+^ CSCs and CD105^-^ TCs)**

| **marker** | **Dendritic cells (CTL DC)** | **CD105^+^ Mo** | **CD105^-^ Mo** |
| --- | --- | --- | --- |
| CD14 | 4.1±0.9 | 2.0±1.0 * | 3.7±3.5 |
| CD83 | 3.6±1.0 | 1.3±0.5 * | 2.0±0.8 |
| CD80 | 26.7±7.9 | 5.3±3.2 *,§ | 12.2±6.8 * |
| CD40 | 11.7±7.0 | 2.3±0.5 * | 2.7±0.9 * |
| α4 integrin | 10.0±4.3 | 3.3±0.5 *,§ | 12.2±8.1 |
| CD54 | 143.8±16.2 | 47.0±7.1 *,§ | 176.0±31.1 |
| α5 integrin | 43.0±9.6 | 6.0±2.5 *,§ | 24.2±2.3 * |
| CD86 | 149.8±66.7 | 15.4±9.4 * | 46.7±25.2 * |
| HLA-DR | 317.1±64.9 | 20.6±8.9 *,§ | 113.3±30.3 * |
| CD1a | 65.0±23.1 | 30.6±16.8 *,§ | 107.8±24.8 |

* P< 0.05 renal cancer cells (CD105^+^ CSCs and CD105^-^ TCs) Mo versus CTL DC

§ P< 0.05 CD105^+^ Mo versus CD105^-^ Mo
