# Supplementary material for: Role of HLA-G and extracellular vesicles in renal cancer stem cell-induced inhibition of dendritic cell differentiation
Source: BMC Cancer. 2015 Dec 24;15:1009. doi: 10.1186/s12885-015-2025-z (PMC4690241; doi:10.1186/s12885-015-2025-z)
Supplement: Additional 2: Table S2. — Mean Fluorescence Intensity (MFI) of monocyte-derived cells stimulated with or without EVs shed by CD105+ CSCs and CD105- TCs. (DOCX 14 kb) [file 12885_2015_2025_MOESM2_ESM.docx]

**Additional Table 2**

**Mean Fluorescence Intensity (MFI) of monocyte-derived cells stimulated with or without EVs shed by CD105^+^ CSCs and CD105^-^ TCs.**

| **marker** | **Dendritic cells (CTL DC)** | **CD105^+^ EV Mo** | **CD105^-^ EV Mo** |
| --- | --- | --- | --- |
| CD83 | 3.6±1.0 | 2.0±1.0 | 3.0±01.7 |
| CD80 | 26.7±7.9 | 9.0±3.6 *,§ | 33.6±27.9 |
| CD40 | 11.7±7.0 | 2.0±0.5 * | 4.0±0.9 * |
| CD54 | 528.0±16.2 | 195.0±7.1 *,§ | 470.8±31.1 |
| α5 integrin | 43.0±9.6 | 15.5±2.5 * | 36.7±2.3 |
| CD86 | 149.8±66.7 | 20.6±9.4 *,§ | 98.0±25.2 |
| HLA-DR | 317.1±64.9 | 36.3±8.9 *,§ | 289.2±30.3 |

* P< 0.05 EV Mo versus CTL DC

§ P< 0.05 CD105^+^ EV Mo versus CD105^-^ EV Mo
