# Supplementary material for: Role of HLA-G and extracellular vesicles in renal cancer stem cell-induced inhibition of dendritic cell differentiation
Source: BMC Cancer. 2015 Dec 24;15:1009. doi: 10.1186/s12885-015-2025-z (PMC4690241; doi:10.1186/s12885-015-2025-z)

**Additional Figure Legend.**

**Additional Figure 1. EVs characterization.**

A. Representative size distribution of EVs shed by CD105^+^ CSCs and CD105^-^ TCs obtained using Nanosight LM10 instrument equipped with the nanoparticle tracking analyses (NTA) 2.0 analytic software. B. Representative cytofluorimetric analysis performed by Guava easyCyte Flow Cytometer of EVs shed by CD105^+^ CSCs and CD105^-^ TCs and analyzed with InCyte software. The following markers were evaluated: CD44, CD105, α5 integrin, α6 integrin, CD73, CD29, CD90 and CD146.


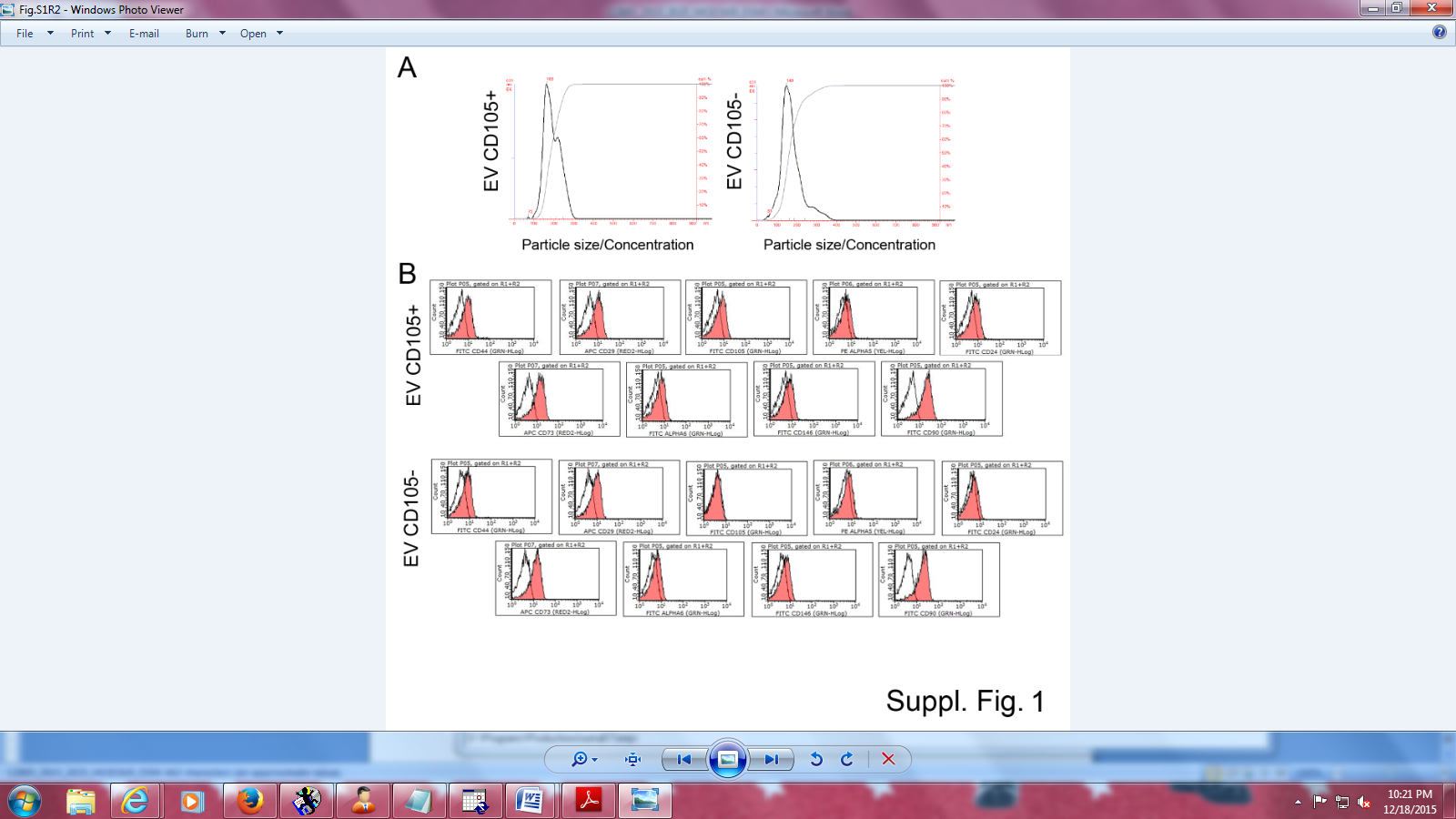

Supplement: Additional 3: Figure S1. — EVs characterization. A. Representative size distribution of EVs shed by CD105+ CSCs and CD105- TCs obtained using NanoSight LM10 instrument equipped with the nanoparticle tracking analysis (NTA) 2.0 analytic software. B. Representative cytofluorimetric analysis performed by Guava easyCyte Flow Cytometer of EVs shed by CD105+ CSCs and CD105- TCs and analyzed with InCyte software. The following markers were evaluated: CD44, CD105, α5 integrin, α6 integrin, CD73, CD29, CD90 and CD146. (DOCX 451 kb) [file 12885_2015_2025_MOESM3_ESM.docx]
